# Effects of metabolic syndrome and its components on pulmonary function and functional capacity in children and adolescents with obesity

**DOI:** 10.1007/s00431-025-06446-5

**Published:** 2025-09-06

**Authors:** Lertluksana Limkul, Kanokporn Udomittipong, Pawinee Charoensittisup, Khunphon Mahoran

**Affiliations:** Division of Pulmonology, Department of Pediatrics, Faculty of Medicine Siriraj Hospital, Mahidol University, 2 Wanglang Road, Bangkok Noi, Bangkok, 10700 Thailand

**Keywords:** 6-MWT, Functional capacity, Metabolic syndrome, Obesity, Pulmonary function

## Abstract

Obesity is a pervasive global health issue frequently associated with metabolic syndrome (MetS). Limited data exist regarding the impact of MetS and its individual components on pulmonary function in obese pediatric populations. This study investigated the relationship between MetS and lung function, and further identified specific MetS components that adversely affect pulmonary outcomes. We enrolled obese children and adolescents aged 7‒18 years. Anthropometric measurements and metabolic assessments were performed. All participants underwent spirometry and the six-minute walk test (6-MWT). Based on MetS criteria, participants were classified into MetS or non-MetS groups. Between-group comparisons were conducted, and regression analyses were used to identify MetS components predictive of lung function and exercise capacity. A total of 155 participants were evaluated (37 [23.9%] with MetS; 118 [76.1%] without). Those with MetS demonstrated a significantly lower 6-MWT distance z-score (‒0.61 ± 1.1 vs ‒0.07 ± 0.99; *P* = 0.006). No significant group differences were found in spirometric parameters. Obesity indices exerted a stronger negative effect on lung function than MetS status. Abdominal circumference (b = ‒0.03, *P* < 0.001) and elevated FBS (> 100 mg/dL; b = ‒0.50, *P* = 0.04) were associated with reduced 6-MWT distance z-scores; they also negatively influenced FEV1% predicted (abdominal circumference: b = ‒0.15, *P* < 0.04; elevated FBS: b = ‒9.04, *P* = 0.02).

*Conclusions*: Obese children and adolescents with MetS show significantly diminished functional capacity as evidenced by lower 6-MWT performance. Among MetS components, increased abdominal circumference and elevated FBS (> 100 mg/dL) emerged as critical predictors of decreased functional capacity.

**Table:** 

**What is Known:** • *Obesity is associated with metabolic syndrome (MetS) and is known to impair lung function.* • *MetS is linked to metabolic and cardiovascular complications, but its impact on pulmonary function in obese pediatric populations remains unclear.*	
**What is New:** • *Obese children and adolescents with MetS have significantly lower functional capacity, as demonstrated by reduced six-minute walk test (6-MWT) performance.* • *Among MetS components, increased abdominal circumference and elevated fasting blood sugar > 100 mg/dL were identified as key predictors of reduced functional capacity and lung function.*

**What is Known:**

• *Obesity is associated with metabolic syndrome (MetS) and is known to impair lung function.*

• *MetS is linked to metabolic and cardiovascular complications, but its impact on pulmonary function in obese pediatric populations remains unclear.*

**What is New:**

• *Obese children and adolescents with MetS have significantly lower functional capacity, as demonstrated by reduced six-minute walk test (6-MWT) performance.*

• *Among MetS components, increased abdominal circumference and elevated fasting blood sugar > 100 mg/dL were identified as key predictors of reduced functional capacity and lung function.*

## Introduction

Obesity is a significant global health concern, driven by its rising worldwide prevalence [1], as well as its adverse effects on multiple organ systems. These effects include metabolic complications, cardiovascular disorders, and respiratory conditions such as obstructive sleep apnea, asthma, exercise intolerance, and reduced pulmonary function [1–3].

Metabolic syndrome (MetS) is a frequent complication of obesity, defined by abdominal obesity plus at least two of the following factors: elevated plasma glucose, elevated triglycerides, low high-density lipoprotein (HDL) cholesterol, or hypertension. This definition follows the 2007 International Diabetes Federation criteria [4]. MetS is recognized as a major risk factor for cardiovascular disease and type 2 diabetes [5], both of which increase morbidity and mortality. It is also an independent risk factor for abnormal respiratory symptoms and reduced lung function [6–9].

Previous studies in obese adults have reported an association between MetS and impaired lung function, specifically reduced forced vital capacity (FVC) [10–13] and forced expiratory volume in 1 s (FEV1) [13]. However, most data on the impact of MetS in obesity focus on adults. Information on obese pediatric populations remains limited and inconclusive. For example, Forno et al. noted a reduced FEV1/FVC ratio among adolescents in the United States [14]. Kim et al. found lower FEV1, FVC, and FEV1/FVC values among Korean children and adolescents [15]. Nonetheless, to the best of our knowledge, no study has examined the effects of MetS on lung function using the six-minute walk test (6-MWT). This submaximal exercise capacity assessment reflects functional capacity.

Therefore, we aimed to determine the effects of MetS on lung function, including spirometry and the 6-MWT, in obese children and adolescents. We also evaluated which MetS components had the greatest impact on impaired lung function and compared the respective influences of obesity and MetS in this population. Our findings may aid clinical management and improve understanding of MetS-related respiratory pathophysiology in these young patients.

## Methods

### Study population

This cross-sectional study was conducted at the Department of Pediatrics, Faculty of Medicine Siriraj Hospital, Mahidol University, Bangkok, Thailand, from March 2024 to January 2025. Children and adolescents aged 7 to 18 years with a diagnosis of obesity were enrolled. Obesity was defined as a body mass index (BMI) z-score greater than 2, according to World Health Organization criteria [16]. The exclusion criteria comprised any history or diagnosis of pulmonary, cardiac, or neuromuscular diseases, thoracic cage deformity, respiratory tract infection within the past 4 weeks, and inability to perform pulmonary function tests.

The study protocol was thoroughly explained to participants and their legal guardians, and informed consent or assent was obtained before enrollment. The study was conducted in accordance with the Declaration of Helsinki and received ethical approval from the Siriraj Institutional Review Board (reference: Si 130/2024). All methods were performed in accordance with the relevant guidelines and regulations.

Demographic data, including age, sex, body weight, height, BMI, BMI z-score, chest circumference, abdominal circumference, and waist-to-height ratio, were collected. Blood pressure was measured, and blood tests were performed to assess metabolic status. These tests included fasting blood sugar (FBS) and a lipid profile (total cholesterol, triglycerides, HDL cholesterol, and low-density lipoprotein cholesterol). Pulmonary function tests—spirometry and the 6-MWT—were administered by a single, well-trained technician.

### Anthropometric evaluation

Body weight and height were measured using a standard tool (Tanita Corporation, Tokyo, Japan). BMI was calculated by dividing body weight (kg) by height (m2). The BMI was then adjusted for age and sex to derive the BMI z-score, in accordance with World Health Organization growth standards [17]. Chest circumference was measured at the level of the nipples, while abdominal circumference was measured between the inferior margin of the last rib and the iliac crest using a tape measure.

### MetS

MetS was defined according to the 2007 International Diabetes Federation criteria [4]. Diagnosis required central obesity, defined as an abdominal circumference at or above the 90th percentile [18], or exceeding 90 cm for boys and 80 cm for girls based on adult cutoffs [19], plus at least two of the following four risk factors:

- FBS ≥ 100 mg/dL or a diagnosis of type 2 diabetes mellitus.
- Triglyceride level ≥ 150 mg/dL.
- Low HDL-cholesterol:
  - < 16 years: HDL-cholesterol < 40 mg/dL.
  - ≥ 16 years: HDL-cholesterol < 40 mg/dL in males and < 50 mg/dL in females.
- High blood pressure:
  - ≥ 10 years: systolic blood pressure ≥ 130 mmHg and/or diastolic blood pressure ≥ 80 mmHg.
  - < 10 years: systolic and/or diastolic blood pressure ≥ 95th percentile for age, sex, and height [20], or ≥ 130/80 mmHg.

### Spirometry

Spirometry was performed using a Vyntus BODY Plethysmograph (Vyaire Medical, Mettawa, IL, USA), following the American Thoracic Society and European Respiratory Society recommendations [21]. The parameters recorded were FVC, FEV1, the FEV1/FVC ratio, forced expiratory flow at 25%‒75% of vital capacity (FEF 25‒75%), and peak expiratory flow. Except for the FEV1/FVC ratio, all parameters were expressed as percentages of the predicted values based on the 2012 multiethnic global lung function equations [22].

#### 6-MWT

The 6-MWT is a performance-based measure of functional fitness recommended by the American Thoracic Society [23]. Participants were instructed to walk as quickly as possible—without running—for 6 min along a flat, 30-m course marked with a visible cone at the turnaround point. They were encouraged to maintain their pace and were given periodic time reminders. Rest was allowed as needed, and the test was discontinued if participants experienced shortness of breath, chest pain, palpitations, or leg cramps. The total distance covered was measured in meters and reported as a z-score based on reference equations established for Asian children and adolescents [24], reflecting the same ethnicity as our study population.

### Statistical analysis

Data were summarized as mean (SD) or number (%) as appropriate. Continuous variables were compared using Student’s *t*-test, whereas categorical variables were compared using the chi-square test or Fisher’s exact test. Simple Linear regression was performed to assess the association between impaired lung function and both MetS and obesity indices. Multiple Linear regression was applied to determine the effect of each component of MetS on impaired lung function. Age and sex were not included as independent variables in the multiple linear regression model since the 6 MWT z-score was already adjusted for sex, age, and height [24]. The pulmonary function parameters presented in %predicted values were also adjusted for sex, age, and height according to GLI-2012 equations for Southeast Asian populations. [22]. Collinearity among independent variables was assessed using the variance inflation factor (VIF). All statistical analyses were conducted using IBM SPSS Statistics, version 29 (IBM Corp, Armonk, NY, USA).

## Results

A total of 155 participants were enrolled, comprised of 37 (23.9%) with MetS and 118 (76.1%) without MetS. The mean (SD) ages in the MetS and non-MetS groups were 13.0 (2.4) years and 11.8 (2.6) years, respectively. The two groups did not differ significantly in sex distribution. However, participants with MetS had higher rates of morbid obesity. They also exhibited greater obesity indices, including body weight, BMI, BMI z-score, chest circumference, abdominal circumference, hip circumference, and waist-to-height ratio. In addition, the MetS group showed a significantly higher proportion of participants with FBS > 100 mg/dL, elevated triglycerides, reduced HDL cholesterol, dyslipidemia, and hypertension (Table 1).

**Table 1:** Demographic and anthropometric characteristics of participants with and without metabolic syndrome

Demographic data	Mean (SD)		*P* value
	MetS (n = 37)	Non-MetS (n = 118)	
Age (y)	13.0 ± 2.4	11.8 ± 2.6	**0.01**
Male sex, n (%)	29 (78.4)	80 (67.8)	0.30
Morbid obesity, n (%)	25 (67.6)	28 (23.9)	** < 0.001**
Weight (kg)	101.9 ± 30.3	75.0 ± 25.1	** < 0.001**
Height (cm)	161.9 ± 11.2	151.6 ± 13.9	** < 0.001**
BMI (kg/m2)	38.4 ± 8.2	31.8 ± 6.8	** < 0.001**
BMI z-score	4.1 ± 1.1	3.5 ± 1.1	**0.005**
CC (cm)	111.2 ± 16.5	96.2 ± 14.6	** < 0.001**
AC (cm)	116.2 ± 17.3	100.0 ± 16.2	** < 0.001**
HC (cm)	116.1 ± 15.3	101.0 ± 14.5	** < 0.001**
WHR	0.72 ± 0.08	0.66 ± 0.08	**0.001**
FBS (mg/dL)	95.9 ± 30.6	87.9 ± 18.6	**0.04**
FBS > 100 mg/dL, n (%)	9 (24.3)	6 (5.1)	**0.002**
TG (mg/dL)	181.6 ± 115.3	94.3 ± 39.9	** < 0.001**
TG > 150 mg/dL, n (%)	22 (59.5)	8 (6.8)	** < 0.001**
HDL-C (mg/dL)	38.0 ± 6.3	47.4 ± 9.2	** < 0.001**
HDL-C < 40 mg/dL, n (%)	29 (78.4)	18 (15.4)	** < 0.001**
Dyslipidemia, n (%)	35 (94.6)	35 (29.7)	** < 0.001**
Hypertension, n (%)	23 (62.2)	19 (16.1)	** < 0.001**

**Abbreviations:** *AC*, abdominal circumference; *BMI*, body mass index; *CC*, chest circumference; *FBS*, fasting blood sugar; *HC*, hip circumference; *HDL-C*, high-density lipoprotein cholesterol; *MetS*, metabolic syndrome; *TG*, triglycerides; *WHR*, waist circumference-to-height ratio

Data are presented as mean (SD) or n (%), as appropriate

Bold values indicate statistically significant differences (*P* < 0.05)

Regarding pulmonary function, the MetS group walked a mean distance of 12.7 m less than the non-MetS group during the 6-MWT, but this difference did not reach statistical significance. However, after adjusting for sex, age, and height according to reference values [24], the MetS group exhibited a significantly lower 6-MWT z-score (‒0.61 ± 1.12 vs ‒0.07 ± 0.99; *P* = 0.006). No significant between-group differences were found in spirometric parameters (Table 2).

**Table 2:** Comparison of spirometry and 6-min walk test performance between participants with and without metabolic syndrome

Pulmonary function tests	Mean (SD)		*P* value
	MetS (n = 37)	Non-MetS (n = 118)	
6-MWT distance (m)	504.2 ± 60.3	516.9 ± 57.3	0.25
6-MWT z-score	–0.61 ± 1.12	–0.07 ± 0.99	**0.006**
FVC (% predicted)	108.9 ± 13.7	112.0 ± 16.1	0.25
FEV1 (% predicted)	102.4 ± 12.5	106.4 ± 15.1	0.15
FEV1/FVC (%)	83.6 ± 6.6	85.3 ± 7.0	0.18
FEF 25–75% (% predicted)	88.6 ± 20.1	95.0 ± 23.3	0.14

**Abbreviations:** 6-*MWT*, 6-min walk test; *FEF* 25–75, forced expiratory flow rate within 25%–75% of forced vital capacity; *FEV1*, forced expiratory volume in 1 s; *FEV1/FVC*, forced expiratory volume in 1 s to forced vital capacity ratio; *FVC*, forced vital capacity; *MetS*, metabolic syndrome

Data are presented as mean (SD)

Bold values indicate statistically significant differences (*P* < 0.05)

When the negative impact on 6-MWT z-score was assessed, MetS alone (R2 = 0.049) showed a weaker association compared with each obesity index, including abdominal circumference (R2 = 0.248), BMI (R2 = 0.305), BMI z-score (R2 = 0.109), and waist-to-height ratio (R2 = 0.184; Table 3).

**Table 3:** Simple Linear regression analysis of the relationship between metabolic syndrome, obesity indices, and 6-min walk test z-score

MetS component	Pulmonary function parameters 6-MWT z-score		
	*b*	*P* value	R2
MetS	–0.539	**0.006**	0.049
AC (cm)	–0.029	** < 0.001**	0.248
BMI (kg/m2)	–0.076	** < 0.001**	0.305
BMI z-score	–0.300	** < 0.001**	0.109
WHR	–5.271	** < 0.001**	0.184

**Abbreviations:** 6-*MWT*, 6-min walk test; *AC*, abdominal circumference; *BMI*, body mass index; *MetS*, metabolic syndrome; *WHR*, waist-to-height ratio

Bold values indicate statistically significant differences (*P* < 0.05)

Analysis of individual MetS components indicated that higher abdominal circumference (b = ‒0.03, *P* < 0.001) and elevated FBS (> 100 mg/dL; b = ‒0.5, *P* = 0.04) were significant predictors of a reduced 6-MWT z-score. Both abdominal circumference (b = ‒0.15, *P* = 0.04) and elevated FBS (b = ‒9.04, *P* = 0.02) also correlated negatively with FEV1% predicted. Moreover, abdominal circumference alone was significantly associated with a lower 6-MWT distance (b = ‒0.83, *P* = 0.005) and FEV1/FVC ratio (b = ‒0.08, *P* = 0.03; Table 4).

**Table 4:** Multiple linear regression analysis of pulmonary function parameters in relation to metabolic syndrome components in obese children and adolescents

Pulmonary function parameters	FBS ≥ 100 mg/dL		TG ≥ 150 mg/dL		HDL < 40 mg/dL		Hypertension		AC (cm)	
	b	*P*	b	*P*	b	*P*	b	*P*	b	*P*
6-MWT distance (m)	−25.65	0.10	16.10	0.18	−1.03	0.92	7.30	0.52	−0.83	**0.005**
6-MWT z-score	−0.50	**0.04**	0.20	0.31	−0.08	0.66	0.17	0.34	−0.03	** < 0.001**
FVC (% predicted)	−7.93	0.07	−1.83	0.58	4.92	0.09	1.12	0.72	−0.11	0.19
FEV1 (% predicted)	−9.04	**0.02**	−0.07	0.98	3.47	0.20	0.55	0.85	−0.15	**0.04**
FEV1/FVC (%)	−1.46	0.44	1.04	0.49	−1.60	0.22	−0.29	0.83	−0.08	**0.03**
FEF 25–75% (% predicted)	−10.22	0.10	2.46	0.61	−1.89	0.66	−2.62	0.56	−0.16	0.19

**Abbreviations:** *6-MWT*, 6-min walk test; *AC*, abdominal circumference; *FBS*, fasting blood sugar; *FEF* 25–75, forced expiratory flow rate within 25%–75% of forced vital capacity; *FEV1*, forced expiratory volume in 1 s; *FVC*, forced vital capacity; *HDL*, high-density lipoprotein cholesterol; *TG*, triglyceride cholesterol

Bold values indicate statistically significant differences (*P* < 0.05)

## Discussion

Our findings highlight a strong association between MetS and reduced lung function in obese children and adolescents. Specifically, participants with MetS showed a significantly lower 6-MWT z-score than non-MetS. Although MetS status affected 6-MWT performance, obesity indices—such as abdominal circumference, BMI, BMI z-score, and waist-to-height ratio—exerted an even stronger negative impact. Among individual MetS components, elevated abdominal circumference and FBS above 100 mg/dL were the most significant predictors of decreased lung function, influencing both 6-MWT z-score and FEV1% predicted. In addition, abdominal circumference alone was negatively associated with the FEV1/FVC ratio.

To our knowledge, no prior study has examined the relationship between MetS and 6-MWT performance in obese children and adolescents. However, adult studies by Gallardo-Alfaro et al. [25] and Ekman et al. [26] support our findings, showing a significantly shorter 6-MWT distance in patients with MetS. The 6-MWT is a simple, well-tolerated method for assessing functional capacity and an individual’s ability to perform daily activities [23, 27]. In this context, the lower 6-MWT z-scores observed in our MetS group indicate that obese children and adolescents with MetS may face greater difficulties in physical exertion. However, there was no statistically significant difference in the absolute 6-MWT distance between MetS and non-MetS group. This emphasized the importance of adjustment of sex, age, and height into the 6-MWT z-score.

Our study found no significant differences in spirometric parameters between participants with and without MetS. Most studies on this association were performed on adults, and their findings remain inconclusive, although several evidence indicates a greater tendency toward restrictive lung dysfunction in individuals with MetS [9, 28–30]. Research focusing on children and adolescents is more limited—particularly in obese populations—and has yielded inconsistent results. In Korean children and adolescents, Kim et al [15] observed that MetS was associated with lower FEV1% predicted, FVC% predicted, and FEV1/FVC ratio, regardless of obesity status. Another study in adolescents in the United States showed a lower FEV1/FVC ratio among those with MetS [14]. Both studies, however, included participants with and without obesity. By contrast, our investigation specifically targeted obese individuals, a population at high risk for MetS. Discrepancies in the findings may stem from our smaller MetS sample size and the exclusive focus on obese participants.

Most available studies examining the relationship between individual MetS components and lung function were conducted in adults, and their findings remain inconclusive [30–33]. Nonetheless, every MetS component appears to be variably linked to altered lung function [9, 30, 34]. Pediatric research in this area is scarce. In a study of Korean children and adolescents, Kim et al. reported that increased abdominal circumference, systolic blood pressure, fasting glucose, and low high-density lipoprotein cholesterol predicted a lower FEV1/FVC ratio [15]. Our study similarly found that elevated abdominal circumference correlated with a reduced FEV1/FVC ratio, whereas both elevated FBS and abdominal circumference predicted reduced FEV1% predicted and poorer 6-MWT performance. No previous pediatric study has included 6-MWT in evaluating the relationship between MetS components and lung function. However, a Swedish adult study by Ekman et al. showed a shorter 6-MWT distance in participants with MetS and diabetes [26]. Insulin resistance, which is not included in standard MetS criteria, has also been associated with impaired lung function [35–37]. We did not examine insulin resistance in our participants. Future studies should incorporate this measure alongside MetS components. Larger investigations are needed to determine how these factors interact to affect respiratory health in obese children and adolescents.

Our findings underscore the intricate interplay between obesity, MetS, and lung function. Central obesity, characterized by increased abdominal circumference, predominantly compromises lung function via mechanical restriction of the diaphragm and chest wall [9, 38, 39]. Additionally, systemic inflammation arising from visceral adipose tissue–derived cytokines may further diminish respiratory function [9, 40, 41]. While metabolic disorders have also been linked to impaired lung function [9, 30], the pathophysiological mechanisms by which MetS components other than abdominal obesity contribute to respiratory decline remain insufficiently understood. Proposed hypotheses involve systemic inflammation, oxidative stress, and vascular injury [9, 30, 42]. Obesity and MetS are overlapping conditions that interact in complex ways. This interplay is exemplified by the fact that obesity—particularly abdominal obesity—is strongly associated with the development of MetS. Accordingly, MetS is a common complication of obesity and abdominal obesity serves as a major diagnostic criterion for its diagnosis.

Our findings highlight the need for comprehensive obesity management strategies, including targeted weight reduction—particularly abdominal fat—and improved glycemic control, to help preserve lung function and enhance physical performance. Further research should clarify the mechanisms by which MetS and its components affect respiratory health in pediatric populations, given the unique developmental, anatomical, and physiological differences between children and adults.

This study has a few noteworthy limitations. First, its cross-sectional design prevents conclusions about causality between MetS components and lung function. For example, although increased abdominal circumference and elevated fasting blood sugar were associated with poorer functional capacity and lung function, the causation of these associations remains uncertain. Future longitudinal research is needed to clarify the temporal sequence and causal pathways of these relationships and to determine whether these associations persist over time. Second, we did not assess insulin resistance, despite emerging evidence of its influence on lung function. This aspect could be a valuable focus for future research, potentially elucidating the relationship or underlying pathophysiology of metabolic syndrome effect on diminished lung function. Third, the MetS group comprised a relatively small sample, which may limit the generalizability of our findings. This limited subgroup size may have constrained the statistical power to detect significant differences in certain pulmonary outcomes, particularly spirometric indices where no significant group differences were observed. Future studies with larger MetS cohorts are warranted to validate our findings.

In conclusion, obese children and adolescents with MetS demonstrated a marked reduction in functional capacity, as evidenced by their lower 6-MWT z-score. Notably, obesity indices—including BMI, BMI z-score, abdominal circumference, and waist-to-height ratio—showed an even stronger negative effect on physical capacity than MetS status alone. Among MetS components, elevated abdominal circumference and FBS exceeding 100 mg/dL emerged as the most significant predictors of reduced functional capacity and spirometric lung function.

## Data Availability

No datasets were generated or analysed during the current study.
